# Gridlock from Diagnosis to Treatment of Multidrug-Resistant Tuberculosis (MDR-TB) in Tanzania: Illuminating Potential Factors for Possible Intervention

**DOI:** 10.24248/EAHRJ-D-16-00330

**Published:** 2017-03-01

**Authors:** Alphonce A Liyoyo, Scott K Heysell, Riziki M Kisonga, Johnson J Lyimo, Liberate J Mleoh, Beatrice K Mutayoba, Isaack A Lekule, Blandina T Mmbaga, Gibson S Kibiki, Stellah G Mpagama

**Affiliations:** a Kilimanjaro Christian Medical University College, Moshi, Tanzania; b Kilimanjaro Clinical Research Institute, Moshi, Tanzania; c Division of Infectious Diseases and International Health, University of Virginia, Charlottesville, Virginia, USA; d Kibong’oto Infectious Disease Hospital, Kilimanjaro, Tanzania; e National TB and Leprosy Programme, Ministry of Health, Community Development, Gender, Elderly and Children, Dar es Salaam, Tanzania; f East African Health Research Commission, Bujumbura, Burundi

## Abstract

**Settings:**

Kibong'oto Infectious Diseases Hospital, Kilimanjaro, Tanzania

**Objective:**

Characterise multidrug-resistant tuberculosis (MDR-TB)-treated cases during the scaling up of molecular diagnostics using Xpert MTB/RIF and GenoType MTBDRplus

**Design:**

Retrospective cohort study

**Results:**

A total of 223 MDR-TB patients were referred to the Kibong’oto Infectious Disease Hospital from January 2013 through December 2014. Four cities—Dar es Salaam, Mbeya, Mwanza, and Tanga—contributed 144 (65%) of referrals. Of the total referred patients, HIV coinfection was found in 92 (41%) and 180 (81%) had history of previous TB treatment. Molecular drug susceptibility testing (DST) contributed 201 (91%) of referrals and resulted in a shorter time from diagnosis to start of treatment, 30 days (95% confidence interval [CI], 26–37), compared to conventional phenotypic DST, 212 days (95% CI, 151–272; *P*<.001). Molecular DST found higher proportions of MDR-TB children and people living with HIV without prior treatment, 5 (12%) and 24 (56%), respectively, compared to those with previous treatment for TB, 4 (2%) and 68 (38%), respectively. The median CD4 count correspondingly was 131 cells/μl (IQR, 109–131) and 200 cells/μl (IQR, 94–337) for MDR-TB diagnosed by phenotypic and molecular diagnostics (*P*=.70). Despite the more rapid time to treatment initiation among patients diagnosed by molecular DST, treatment outcomes, including time to sputum culture conversion, did not differ compared to those diagnosed with conventional phenotypic DST. Regardless of the method of diagnosis, MDR-TB/HIV coinfected patients who died had lower CD4 counts (mean 86 ± 87 cells/μl) than survivors (mean 274 ± 224 cells/μl; *P*=.02).

**Conclusion:**

Molecular diagnostics appear to speedup the time to treatment initiation, but may not improve other treatment outcomes.

## INTRODUCTION

Multidrug resistance to tuberculosis (MDR-TB) is defined as *Mycobacterium tuberculosis* (MTB) with at least resistance to isoniazid and rifampicin, and is associated with high morbidity and mortality.^1^ Diagnosis of MDR-TB in resource-limited settings is immensely challenging, requiring not only identification of MTB but also drug susceptibility testing (DST) to confirm, at minimum, isoniazid and rifampin resistance. While DST can be performed by conventional phenotypic methods such as the 1% agar proportion, it requires mycobacterial culturing of sputum in biosafety facilities and takes upward of 12 weeks from the time the sputum specimen is submitted to produce results. Alternatively, mutations in the drug resistance-conferring regions of the MTB genome have reasonable diagnostic accuracy, compared to phenotypic DST, for several important TB medications, including isoniazid and rifampin. In the best-case scenario, Xpert MTB/RIF or GenoType MTBDRplus can deliver a result from an uncultured sputum specimen within a 2-day period, often as early as within the same clinic visit. The near immediacy of this faster testing approach has led to commercialised assays and a rather unprecedented global roll out.^2^

We previously highlighted several unique aspects of the first cohort of patients treated for MDR-TB in Tanzania, 2009–2011, the majority of whom were diagnosed using conventional phenotypic DST.^3^ Importantly, the average time from specimen collection to the start of MDR-TB treatment for that cohort was 9 months. At that time, the proportion of patients with MDR-TB with HIV coinfection was only 14%, while the overall country prevalence of HIV in TB notification was 40%. We hypothesised that patients with MDRTB treated in the first cohort were those who were relatively healthy enough to survive the prolonged diagnostic and referral process, a tip of the iceberg, and that many others with MDR-TB either died before referral or the conventional phenotypic method of DST created a barrier of cost, logistical complexity, and lack of availability that led clinicians to send for this testing only when a patient had failed multiple rounds of TB treatment. Supporting these assumptions were data indicating that 86% of cases had been treated for TB at least twice previously, and those with HIV coinfection were relatively immune reconstituted, with the majority on anti-retroviral therapy (ART).^3^ We further hypothesised that an increase in the availability of rapid molecular DST would identify sicker patients before they died—including more of those with HIV and advanced immunosuppression—and proportionally more with primary MDR-TB or no prior TB treatment.

The scale up of molecular diagnostic testing using XpertMTB/RIF and GenoType MTB/DRplus assays began as early as 2011 in Tanzania. A preliminary study found significantly reduced time from specimen submission to MDR-TB treatment to an average of 2 months;^4^ however, the duration was still long compared to the test characteristics and projections from more widespread roll out. This study was limited in that it did not examine the impact of molecular DST on MDR-TB treatment outcomes. Additionally, findings from a large prospective evaluation of Xpert MTB/RIF testing in South Africa showed the testing method did not result in a decrease in mortality or increase in the retention of patients in TB treatment.^5^

In 2014, the World Health Organization (WHO) officially recommended DST using XpertMTB/RIF for all people living with HIV (PLHIV) with presumptive TB regardless of treatment history, thus providing additional advantage for early screening of MDR-TB.^6^ Although Tanzania continues to scale up molecular DST for MDR-TB diagnosis in accordance to global recommendations,^7^ it remains to be seen if this paradigm shift more regionally translates to a change in MDR-TB clinical presentation or treatment outcomes.

To examine this issue, from 2013 to 2014, we had the unique opportunity to characterise consecutive patients with MDR-TB admitted at Kibong’oto Infectious Disease Hospital (KIDH)—the only national center coordinating all MDR-TB treatment—during the country's transition from conventional phenotypic DST to the faster, more specific molecular DST in order to ascertain the effect of molecular diagnostics on these important clinical parameters.

## METHODOLOGY

### Participants

From 1 January 2013 through 31 December 2014, the study team recruited patients with MDR-TB referred from all regions of the country to Kibong’oto Infectious Disease Hospital (KIDH) for MDR-TB treatment. Patients with previously treated MDR-TB and readmitted as failures or relapse or lost to follow-up were excluded. Those suspected of MDR-TB were screened in the domicile regions using molecular diagnostics (Xpert MTB/RIF or GenoType MTBDRplus),^8^ or conventional drug-resistance surveillance performed programmatically at the Central TB Reference Laboratory (CTRL) in Dar es Salaam.

Once diagnosed by a positive test for rifampin resistance, MDR-TB patients were transported to and treated at KIDH with a standardised regimen comprised of at least 4 new anti-TB drugs and pyrazinamide during the inpatient intensive phase.^9^ The four new drugs included 1 injectable agent (kanamycin or capreomycin), 1 flouroquinolone (levofloxacin), and 2 group-4 drugs (ethionamide and cycloserine). Medications were given based on weight and administered under direct observation daily for a period of 8 months. For patients experiencing drug intolerance of any of the group-4 drugs, para-aminosalicylic acid was substituted. Novel or repurposed anti-TB agents, such as bedaquiline, delamanid, clofazimine, and linezolid, were not available at that time.

Prior to initiation of second-line anti-TB drugs, baseline measurements and levels were taken such as height, weight, complete blood count, liver and renal function tests, sputum mycobacterial culture, and HIV serodiagnostics; if the latter was positive, a CD4 count was also tested per hospital routine. Treatment responses were monitored monthly and the patient was considered as culture converted if 2 consecutive negative sputum cultures were collected at least 1 month apart. Thereafter, clinically improved patients were discharged for an additional 12 months of continuation treatment that excluded the injectable agent.

### Design

This retrospective cohort study reviewed consecutive medical charts of MDR-TB patients admitted to KIDH during the study period. Demographic and clinical data were extracted from charts. Treatment outcome variables were comprised of the time from sputum culture conversion to negative (intermediate outcome) and the final treatment response was categorised as either favorable or unfavorable. The predictors of treatment outcome included demographic features, the method of susceptibility testing, time from diagnosis to referral, HIV status, CD4 count, the number of previous TB treatment episodes, and nutrition status.

### Definition and Measures

Patients who had never been exposed to TB drugs or had taken anti-TB drugs for less than a month were classified as primary MDR-TB. Patients with a history of previous TB treatment—with exposure of category I or II treatments for at least 1 month or more—and a recognised World Health Organization (WHO) treatment outcome of cured, treatment complete, treatment failure, relapse, or lost to follow-up, were classified as secondary or acquired MDR-TB.^9^ PLHIV patients were those with a known HIV diagnosis and on anti-retroviral therapy (ART) prior to referral; all other patients were tested for HIV at KIDH at the time of MDR-TB treatment initiation regardless of their prior testing history. MDR-TB treatment outcomes were determined at the conclusion of both intensive and continuation phases of therapy. Favorable outcomes included treatment completed as recommended without evidence of failure with or without consecutive culture taken at least 30 days apart and reported culture negative during the continuation phase. Unfavorable outcomes included death by any cause; lost to follow-up, if treatment was interrupted for a greater than 8 weeks; treatment failure, if treatment was terminated or there was a need for a permanent regimen change of at least 2 anti-TB drugs because of lack of culture conversion by the end of intensive phase; or bacteriological reversion to culture positive in the continuation phase.^10^ The body mass index (BMI) was defined as the ratio of weight (kg)/height^2^ (m^2^) at baseline. Low BMI (<18.5) was further classified into 3 categories: BMI of (17.00–18.49), (16.00–16.99), and (<16) were considered as mild, moderate, and severe malnutrition, respectively, according to WHO criteria.^11^

### Data Quality Assurance and Statistical Analysis

Data were double entered from source documents in Microsoft Excel (Version 14.2.3), then transferred to SPSS (Version 20) for analysis. Descriptive results were conveyed as simple proportion with a percentage, as a mean with 95% confidence interval (CI) or standard deviation (SD), or as a median with interquartile range (IQR), when applicable. Proportions were compared using a chi-square test, while means were compared using an independent t-test and medians using the Mann Whitney U test for non-parametric data. Determinants of final treatment outcome were examined using logistic regression with HIV status; CD4 count, in cases coinfected with HIV; age; gender; history of previous TB treatment; and nutrition status as predictors. All statistical tests were two-tailed with a *P* value of <.05 considered as significant.

### Ethical Approval

The Kilimanjaro Christian Medical University College Research Ethics and Review Committee (CRERC), the KIDH management, and the University of Virginia approved this study.

## RESULTS

### Demographics and Clinical Characteristics

A total of 223 patients with MDR-TB were referred to KIDH from January 2013 to December 2014. Four (17%) regions that include 4 large cities—Dar es Salaam, Mbeya, Mwanza, and Tanga—contributed 144 (65%) of MDR-TB referrals. Although the mean age was 38 ± 15 years, children under 12 years were only 9 (4%). Men constituted 145 (65%) of referrals and 92 (41%) had HIV coinfection with median CD4 count of 200 cells/μl (IQR). History of previous TB treatment was found in 180 (81%) of the study population, 121 (76%) of whom had at least 2 prior treatment episodes. The MDR-TB diagnosis in 204 (91%) was through the molecular diagnostic tools, the vast majority 184 (83%) by Xpert MTB/RIF (Table 1).

**TABLE 1.** Distributions of Demographic and Clinical Characteristics of Patients with MDR-TB Referred for Treatment (N=223).

Characteristics	Subcategories	Number
Age, years, mean (SD)	NA	38 (15)
Paediatric, No. (%)	Under 5 years	4 (1.8)
	5–12 years	5 (2.2)
Sex, No. (%)	Male	145 (65)
	Female	78 (35)
HIV status, No. (%)	Negative	129 (58)
	Positive	92 (41)
	Unknown	2 (1)
CD4 count for PLHIV, median (25–75 IQR)	NA	200 (99, 333)
Region of referral/domicile, No. (%)	Dar es Salaam	99 (44)
	Mwanza	23 (10)
	Tanga	11 (5)
	Mbeya	11 (5)
	Others^a^	79 (37)
History of PTB treatment, No. (%)	Yes	180 (81)
	No	43 (19)
Number of PTB episodes, No. (%)	1	59 (33)
	2	84 (47)
	3	27 (15)
	4 or more	10 (5)
Method of drug susceptibility test, No. (%)	Conventional	19 (9)
	Molecular	204 (91)
Type of molecular DST, No. (%)	GenoType MTB/RIF	184
	GenoType MTBDRplus	19
Duration from specimen submission to treatment, days, mean (95% CI)	Conventional	210 (150, 270)
	GenoType MTB/RIF	27 (20, 30)
	GenoType MTBDRplus	70 (40, 100)
Time to culture conversion, No. (%)	3 months or less	166 (72)
	More than 3 months	27 (12)
	Unknown	37 (16)
End of MDR-TB treatment outcomes, No. (%)	Favorable	179 (80)
	Unfavorable	44 (20)
Unfavorable treatment outcomes, No. (%)	Died	34 (15)
	Defaulted	6 (3)
	Reverted	3 (1)
	Unknown	1 (<1)

Abbreviations: CI, confidence interval; DST, drug susceptibility testing; IQR, interquartile range; MDR-TB, multidrug-resistant tuberculosis; PLHIV, people living with HIV; SD, standard deviation; TB, tuberculosis.

a Others refer to Iringa, Morogoro, Kilimanjaro, Tabora, Tanga, Mara, Dodoma, Pwani, Kagera, Geita, Ruvuma, Singida, Manyara, Arusha, Mtwara, Rukwa, Lindi, Shinyanga, Zanzibar, and Kigoma.

### Effect of the DST Methods on the Presenting MDR-TB Features

For patients diagnosed with the conventional phenotypic DST, the mean time from diagnosis to MDR-TB treatment initiation was 212 days (95% CI, 151–272), compared to those diagnosed with the molecular DST method that had a mean time of 31 days (95% CI, 26–37; *P*<.001) (Table 2). Interestingly, patients diagnosed with Xpert MTB/RIF had a shorter duration to treatment initiation 27 days (IQR, 20–30) than those diagnosed by GenoType MTBDRplus (70 days, IQR, 40–100). All 19 patients diagnosed with conventional phenotypic DST had a previous history of TB treatment (100%), compared to 161 (79%) of those diagnosed by molecular DST (*P*=.03). Furthermore, 89 (44%) of PLHIV were diagnosed by molecular DST compared to only 3 (16%) diagnosed by phenotypic DST (*P*= .02).

**TABLE 2.** Comparison of the Clinical Characteristics of Patients with Patients with MDR-TB Referred After Diagnosis with Molecular and Conventional Methods

Characteristics	Phenotypic DST (N=19)	Molecular DST (N=204)	*P* Value
Age, years, mean (SD)	34 (13)	38 (15)	.29
Sex, male, No. (%)	10 (53)	135 (66)	.30
Paediatric, No. (%)	0 (0)	9 (100)	.99
HIV status, No. (%)	3 (16)	89 (44)	.02
CD4 count for PLHIV, median (IQR)	131 (109, 131)	200 (94, 337)	.70
History of TB treatment, No. (%)	19 (100)	161 (79)	.03
Episodes of TB treatment, median (IQR)	2 (2, 3)	2 (1, 2)	.22
Duration from diagnosis to treatment, days, mean (95% CI)	212 (151, 272)	30 (26, 37)	<.001
Time to culture conversion after month 3, No. (%)	4 (25)	23 (13)	.19
Unfavorable treatment outcomes, No. (%)	5 (26)	39 (19)	.54

Abbreviations: CI, confidence interval; DST, drug susceptibility testing; IQR, interquartile range; MDR-TB, multidrug-resistant tuberculosis; PLHIV, people living with HIV; SD, standard deviation; TB, tuberculosis.

Given the increased proportion of primary MDR-TB patients diagnosed with molecular DST compared to historical norms using only phenotypic DST, we further examined the clinical characteristics of primary and previously treated MDR-TB patients. A higher proportion (12%) of children (<12 years) were classified as primary MDR-TB compared to those with prior treatment (2%) (*P*=.02). Additionally, PLHIV comprised a higher proportion in primary MDR-TB cases, 24 (56%), than in previously treated cases, 68 (38%) (*P*=.03), although CD4 counts did not vary between those groups (Table 2). Despite primary MDR-TB having a non-significantly shorter time from sputum collection to start of MDR-TB treatment—likely due to their mode of diagnosis—history of previous TB treatment did not comparatively prolong time to culture conversion (*P*=.11) or lead to a greater proportion of unfavorable treatment outcomes (*P*=.6) in other patients (Table 3).

**TABLE 3.** Comparison of Clinical–Demographic Characteristics of Patients with MDR-TB With and Without History of Previous TB Treatment

Characteristics	Primary MDR-TB (N=43)	Acquired or Secondary MDR-TB (N=180)	*P* Value
Age, years, mean (SD)	36 (15)	38 (14)	.38
Sex, male, No. (%)	22 (51)	123 (68)	.49
Paediatric, No. (%)	5 (12)	4 (2)	.02
HIV status, No. (%)	24 (56)	68 (38)	.03
CD4 count for PLHIV, median (IQR)	161 (80, 308)	209 (99, 367)	.27
Duration from diagnosis to treatment, days, mean (95% CI)	27 (19, 34)	53 (41, 64)	.51
Time to culture conversion after month 3, No. (%)	2 (5)	24 (16)	.11
Unfavorable treatment outcomes, No. (%)	7 (16)	37 (21)	.60

Abbreviations: CI, confidence interval; DST, drug susceptibility testing; IQR, interquartile range; MDR-TB, multidrug-resistant tuberculosis; PLHIV, people living with HIV; SD, standard deviation; TB, tuberculosis. 2 MDR-TB patients had unknown HIV status.

### Comparison of Clinical-Demographic Factors with Final Treatment Outcomes

Twenty-seven patients (12%) had delayed culture conversion (>3 months); however, for 37 (16%) of the patients time to culture conversion could not be estimated. In follow-up, 44 (20%) had unfavorable treatment outcomes, which included death in 33 (77%) and culture reversion to positive in 3 (7%) (Table 1). The latter yielded 1 (2%) extensively drug-resistant (XDR)-TB case. Of the patients who died, 15 (44%) were within 2 months of MDR-TB treatment initiation. Logistic regression analysis of the association of final treatment outcomes with potential covariates such as age, gender, HIV, CD4 count, and history of previous TB treatment was performed; only CD4 count had statistical significance in predicting unfavorable treatment outcomes (*P*=.02). The mean CD4 count of MDR-TB patients who died was 86 ± 87 cells/μl compared to those with favorable outcome that had a mean CD4 count of 274 ± 224 cells/μl (Table 4).

**TABLE 4.** Comparison of Patients Who Completed MDR-TB Treatment to Those Who Died

Characteristics	Classification	Favorable Outcomes (N=179)	Died (N=34)	*P* Value
Age, years, mean (SD)	N/A	37 (13)	41 (20)	.6
Duration from diagnosis to treatment, mean (95% CI)	N/A	48 (37, 59)	42 (19, 65)	.4
Sex, No. (%)	Male	113 (64)	26 (76)	.4
	Female	65 (36)	9 (24)	
HIV status, No. (%)	Positive	75 (84)	13 (15)	.3
	Negative	102 (88)	21 (16)	
CD4 count for PLHIV, mean (SD)	N/A	274 (224)	86 (87)	.02
History PTB treatment, No. (%)	Yes	142 (79)	30 (17)	.27
	No	35 (83)	5 (12)	
Episodes of TB treatment episodes, mean (SD)	N/A	2 (1)	2 (1)	.23
Nutrition status, No. (%)	Normal	62 (90)	4 (6)	.28
	Mild malnutrition	19 (83)	3 (13)	
	Moderate malnutrition	9 (64)	4 (29)	
	Severe malnutrition	26 (79)	5 (15)	

Abbreviations: N/A, Not applicable, CI, confidence interval; MDR-TB, multidrug-resistant tuberculosis; PLHIV, people living with HIV; SD, standard deviation; TB, tuberculosis.

## DISCUSSION

Following the roll out of molecular diagnostics for MDR-TB in Tanzania, we expectedly found more cases of MDR-TB with HIV coinfection, paediatric MDR-TB, and primary MDR-TB referred for treatment initiation. However, we were surprised to discover little difference in treatment outcomes between patients diagnosed by molecular or phenotypic methods. This observation could be for a few reasons. While the molecular tests may speed treatment initiation, the original hypothesis claiming molecular tests would reduce early mortality from MDR-TB may have been overstated. Our findings support those of a large cohort in South Africa where the roll out of Xpert MTB/RIF did not improve TB treatment outcomes.^12^ Instead, outcomes may depend on other factors such as enrollment in and adherence to HIV ART, use of second-line anti-TB DST, and other host factors, including pharmacokinetic variability. We still found delays of nearly 1 month on average from diagnosis with a molecular test to treatment initiation; however, if those delays could be further reduced then a treatment outcome benefit may be observed. Also, of those with prior TB treatment, comparatively few were diagnosed by the conventional phenotypic DST, and thus may have other uncharacterised reasons for survival.

While it still possible that molecular diagnostics may prevent mortality, this benefit will be difficult to quantify in the Tanzanian setting without a cluster randomised trial, which may now be difficult to study given the widespread attempt to roll out Xpert MTB/RIF. Interestingly, compared to the GenoType MTBDRplus used in our settings, Xpert MTB/RIF reduced delay of treatment initiation by 2.5 fold. The estimated time for GenoType MTBDRplus was 70 days, which is slightly worse than 55 days reported in South Africa,^13^ while the delay for Xpert MTB/RIF was 27 days, compared to 26 in similar settings and 4 days in other well-resourced settings.^12,14^ These differences may be explained by the hospital laboratories that use GenoType MTBDRplus compared to Xpert MTB/RIF or other factors not tested, including specimen transport time and the means of relaying results back to patients and providers.^15^ Also, the GenoType MTBDRplus suffers additional delay of testing sputum specimen on smear results prior to test.^16^ Regardless of the test type, the official policy of the Tanzania National TB and Leprosy Programme accepts health system delays of no more than 14 days, yet this research suggests that considerably longer delays still exist. We are currently undertaking a nationwide study to investigate patient- and provider-related and health system-associated factors to understand the magnitude of these delays on disease transmission and to inform the design of strategies for intervention.

In Tanzania, we have observed a decline in the well-known risk factor for MDR-TB—the proportion of patients with MDR-TB categorised as having multiple episodes of retreatment for drug-susceptible TB—which was initially as high as 86% and is now down to 67%.^3,4^ This statistic can be used as a crude marker for how widespread DST for MDR-TB has matured throughout the country. Indeed, in 2014 it was estimated that only 9,506 (40%) of new and 882 (34%) retreatment cases were screened for MDR-TB in 2014. Regardless of methodology, phenotypic DST or one of the molecular methods, these proportions in new and retreatment cases seem woefully low. Continued evidence points to the fact that even in the setting of adequate adherence, patients with prior TB treatment failure are at risk for acquiring drug resistance largely due to suboptimal serum drug exposure originating from the individual's pharmacokinetic variability.^17^ While a rare event, this phenomenon occurs more often among those presenting with prior TB treatment failure. This hypothesis is further supported by several studies in Tanzania that found very low serum drug levels to first-line anti-TB drugs in a noteworthy proportion of patients.^18–20^ To prevent the emergence of MDR-TB or more complex forms of resistance, therapeutic drug monitoring (TDM) for optimal treatment could be beneficial.^21^ We support expert recommendations of applying TDM in resource-limited settings to individualise dosing to ensure adequate pharmacokinetic/pharmacodynamic (PK/PD), speed the time to culture conversion, decrease relapse, and prevent the development of resistance.^22,23^ Barriers of cost and expertise may be overcome by using more field-appropriate methods, such as the use of capillary dried blood spots that do not require cold storage. When compared to the health system effort necessary to implement a molecular diagnostics strategy for MDR-TB diagnosis, TDM may be a life-saving complementary strategy.^24–26^

Lastly, this cohort has a remarkable high number of new MDR-TB in children and PLHIV compared to those previously treated for TB. A recent systematic review confirmed that HIV infection is an independent risk factor for MDR-TB, emphasising several risk factors including defects in infection prevention control, especially in hospital settings.^27^ Therefore, routine screening of PLHIV to identify and test patients presenting with at least one of the signs and symptoms of MDR-TB—such as current cough, fever, weight loss, or night sweats—should eventually impact morbidity and mortality in this group.^6^ To achieve the STOP TB Partnership global plan of 90(90)90—to diagnose least of 90% of all the people with TB, reach 90% of key populations (the most vulnerable or under-served), and achieve 90% treatment success—then diligent approaches are needed for MDR-TB identification in currently underserved populations, such as children.^28^ Estimates have shown that every year there is an enormous detection gap for children with not only TB but also MDR-TB.^29^ Several challenges surround diagnosis of MDR-TB in children; the paucibacillary nature of TB and the inability of paediatric patients to produce an adequate sputum specimen complicate diagnostic processes. Further operational research that combines available diagnostic strategies, such as active specimen collection in children using sputum induction or gastric aspiration, and tests a wide variety of diagnostics in different settings and age groups may begin to address this diagnostic gap.

Despite the inclusion of all patients treated for MDR-TB in Tanzania during the study period, there were limitations to the analyses, as the hospital-based observational approach included only those who were successfully referred to KIDH for MDR-TB treatment and excluded patients unable to reach the MDR-TB facility due to early death and loss to follow-up during the diagnosis process.

In summary, we have observed a clear shift in the use of molecular diagnostics for referral to MDR-TB treatment in Tanzania, but that shift has not been associated with obvious improvements in MDR-TB treatment outcomes. Molecular diagnostics appear, however, to have contributed to an earlier detection, diagnosis, and treatment of MDR-TB in children and PLHIV. However, despite changes in diagnostic practices, delays persist and should be considerably reduced. As such, we propose MDR-TB diagnostic strategies include a community education component that explains the factors contributing to diagnostic delay for both patients and health systems. Alternative strategies to prevent MDR-TB may also be necessary; and, once MDR-TB is diagnosed, further actions to address MDR-TB treatment failure—such as the use of quantitative second-line DST and individualised regimens—mayultimatelyleadtothefullpotentialgainsenvisioned for the roll out of molecular MDR-TB diagnostics.
